# Supplementary material for: Unveiling the Bioactive Potential of the Invasive Jellyfish Phyllorhiza punctata Through Integrative Transcriptomic and Proteomic Analyses
Source: Biomolecules. 2025 Aug 4;15(8):1121. doi: 10.3390/biom15081121 (PMC12383608; doi:10.3390/biom15081121)
Supplement: Supplementary file 1 [file biomolecules-15-01121-s001.zip › Suplementary Material/FileS3/PEPFOLD4/XP_065071306_16831/clusters.html]

 PEP-FOLD clusters


Download archive of all models  here   

|  |  |  |
| --- | --- | --- |
| # | file | sOPEP |
| # cluster 1 - Size: 9 |  |  |
|  | XP\_065071306\_16831-model1 | -25.687 |
|  | XP\_065071306\_16831-model1.1 | -25.5983 |
|  | XP\_065071306\_16831-model1.2 | -25.423 |
|  | XP\_065071306\_16831-model1.3 | -25.4095 |
|  | XP\_065071306\_16831-model1.4 | -25.4074 |
|  | XP\_065071306\_16831-model1.5 | -25.366 |
|  | XP\_065071306\_16831-model1.6 | -25.324 |
|  | XP\_065071306\_16831-model1.7 | -25.2771 |
|  | XP\_065071306\_16831-model1.8 | -25.0513 |
| # cluster 2 - Size: 27 |  |  |
|  | XP\_065071306\_16831-model2 | -22.0614 |
|  | XP\_065071306\_16831-model2.1 | -22.0268 |
|  | XP\_065071306\_16831-model2.2 | -21.9861 |
|  | XP\_065071306\_16831-model2.3 | -21.9818 |
|  | XP\_065071306\_16831-model2.4 | -21.9692 |
|  | XP\_065071306\_16831-model2.5 | -21.919 |
|  | XP\_065071306\_16831-model2.6 | -21.8881 |
|  | XP\_065071306\_16831-model2.7 | -21.8431 |
|  | XP\_065071306\_16831-model2.8 | -21.8172 |
|  | XP\_065071306\_16831-model2.9 | -21.7847 |
|  | XP\_065071306\_16831-model2.10 | -21.7648 |
|  | XP\_065071306\_16831-model2.11 | -21.7536 |
|  | XP\_065071306\_16831-model2.12 | -21.735 |
|  | XP\_065071306\_16831-model2.13 | -21.6516 |
|  | XP\_065071306\_16831-model2.14 | -21.5431 |
|  | XP\_065071306\_16831-model2.15 | -21.4948 |
|  | XP\_065071306\_16831-model2.16 | -21.4775 |
|  | XP\_065071306\_16831-model2.17 | -21.4767 |
|  | XP\_065071306\_16831-model2.18 | -21.4757 |
|  | XP\_065071306\_16831-model2.19 | -21.3549 |
|  | XP\_065071306\_16831-model2.20 | -21.3305 |
|  | XP\_065071306\_16831-model2.21 | -21.3194 |
|  | XP\_065071306\_16831-model2.22 | -20.9902 |
|  | XP\_065071306\_16831-model2.23 | -20.8392 |
|  | XP\_065071306\_16831-model2.24 | -20.7046 |
|  | XP\_065071306\_16831-model2.25 | -20.6145 |
|  | XP\_065071306\_16831-model2.26 | -20.5735 |
| # cluster 3 - Size: 44 |  |  |
|  | XP\_065071306\_16831-model3 | -20.3156 |
|  | XP\_065071306\_16831-model3.1 | -20.18 |
|  | XP\_065071306\_16831-model3.2 | -19.7815 |
|  | XP\_065071306\_16831-model3.3 | -19.7151 |
|  | XP\_065071306\_16831-model3.4 | -19.6319 |
|  | XP\_065071306\_16831-model3.5 | -19.5796 |
|  | XP\_065071306\_16831-model3.6 | -19.5777 |
|  | XP\_065071306\_16831-model3.7 | -19.5254 |
|  | XP\_065071306\_16831-model3.8 | -19.2872 |
|  | XP\_065071306\_16831-model3.9 | -19.2764 |
|  | XP\_065071306\_16831-model3.10 | -19.2601 |
|  | XP\_065071306\_16831-model3.11 | -19.2481 |
|  | XP\_065071306\_16831-model3.12 | -19.1708 |
|  | XP\_065071306\_16831-model3.13 | -19.0714 |
|  | XP\_065071306\_16831-model3.14 | -19.0392 |
|  | XP\_065071306\_16831-model3.15 | -19.0339 |
|  | XP\_065071306\_16831-model3.16 | -18.9959 |
|  | XP\_065071306\_16831-model3.17 | -18.9782 |
|  | XP\_065071306\_16831-model3.18 | -18.9618 |
|  | XP\_065071306\_16831-model3.19 | -18.9249 |
|  | XP\_065071306\_16831-model3.20 | -18.8647 |
|  | XP\_065071306\_16831-model3.21 | -18.8316 |
|  | XP\_065071306\_16831-model3.22 | -18.8245 |
|  | XP\_065071306\_16831-model3.23 | -18.8159 |
|  | XP\_065071306\_16831-model3.24 | -18.8032 |
|  | XP\_065071306\_16831-model3.25 | -18.8 |
|  | XP\_065071306\_16831-model3.26 | -18.7874 |
|  | XP\_065071306\_16831-model3.27 | -18.7533 |
|  | XP\_065071306\_16831-model3.28 | -18.7351 |
|  | XP\_065071306\_16831-model3.29 | -18.712 |
|  | XP\_065071306\_16831-model3.30 | -18.6804 |
|  | XP\_065071306\_16831-model3.31 | -18.6149 |
|  | XP\_065071306\_16831-model3.32 | -18.603 |
|  | XP\_065071306\_16831-model3.33 | -18.5792 |
|  | XP\_065071306\_16831-model3.34 | -18.5644 |
|  | XP\_065071306\_16831-model3.35 | -18.5628 |
|  | XP\_065071306\_16831-model3.36 | -18.5624 |
|  | XP\_065071306\_16831-model3.37 | -18.4977 |
|  | XP\_065071306\_16831-model3.38 | -18.4719 |
|  | XP\_065071306\_16831-model3.39 | -18.4488 |
|  | XP\_065071306\_16831-model3.40 | -18.4096 |
|  | XP\_065071306\_16831-model3.41 | -18.3904 |
|  | XP\_065071306\_16831-model3.42 | -18.0839 |
|  | XP\_065071306\_16831-model3.43 | -17.9359 |
| # cluster 4 - Size: 13 |  |  |
|  | XP\_065071306\_16831-model4 | -19.8235 |
|  | XP\_065071306\_16831-model4.1 | -19.0485 |
|  | XP\_065071306\_16831-model4.2 | -18.8938 |
|  | XP\_065071306\_16831-model4.3 | -18.5559 |
|  | XP\_065071306\_16831-model4.4 | -18.5008 |
|  | XP\_065071306\_16831-model4.5 | -18.3058 |
|  | XP\_065071306\_16831-model4.6 | -18.2769 |
|  | XP\_065071306\_16831-model4.7 | -18.2216 |
|  | XP\_065071306\_16831-model4.8 | -18.2178 |
|  | XP\_065071306\_16831-model4.9 | -18.1916 |
|  | XP\_065071306\_16831-model4.10 | -18.1066 |
|  | XP\_065071306\_16831-model4.11 | -18.0464 |
|  | XP\_065071306\_16831-model4.12 | -17.7735 |
| # cluster 5 - Size: 5 |  |  |
|  | XP\_065071306\_16831-model5 | -18.6515 |
|  | XP\_065071306\_16831-model5.1 | -18.6168 |
|  | XP\_065071306\_16831-model5.2 | -18.4871 |
|  | XP\_065071306\_16831-model5.3 | -18.4696 |
|  | XP\_065071306\_16831-model5.4 | -18.4048 |
| # cluster 6 - Size: 2 |  |  |
|  | XP\_065071306\_16831-model6 | -18.3779 |
|  | XP\_065071306\_16831-model6.1 | -18.154 |
|  |  |  |
